# Identification of Human Leukotriene A4 Hydrolase Inhibitors Using Structure-Based Pharmacophore Modeling and Molecular Docking

**DOI:** 10.3390/molecules25122871

**Published:** 2020-06-22

**Authors:** Suaad A. Audat, Nizar A. Al-Shar’i, Buthina A. Al-Oudat, Amanda Bryant-Friedrich, Mel F. Bedi, Aref L. Zayed, Qosay A. Al-Balas

**Affiliations:** 1 Department of Chemistry, College of Science and Arts, Jordan University of Science and Technology, P.O. Box 3030, Irbid 22110, Jordan; 2 Department of Medicinal Chemistry and Pharmacognosy, Faculty of Pharmacy, Jordan University of Science and Technology, P.O. Box 3030, Irbid 22110, Jordan; nashari@just.edu.jo (N.A.A.-S.); baoudat@just.edu.jo (B.A.A.-O.); alzayed@just.edu.jo (A.L.Z.); qabalas@just.edu.jo (Q.A.A.-B.); 3 Department of Medicinal and Biological Chemistry, College of Pharmacy and Pharmaceutical Sciences, University of Toledo, Toledo, OH 43606, USA; Amanda.Bryant-Friedrich@utoledo.edu (A.B.-F.); Fernand.Bedi@utoledo.edu (M.F.B.)

**Keywords:** leukotriene A4 hydrolase, leukotriene B4, anti-cancer and anti-inflammatory agents, pharmacophore modeling, moelcular docking

## Abstract

Leukotriene B4 (LTB4) is a potent, proinflammatory lipid mediator implicated in the pathologies of an array of inflammatory diseases and cancer. The biosynthesis of LTB4 is regulated by the leukotriene A4 hydrolase (LTA_4_H). Compounds capable of limiting the formation of LTB4, through selective inhibition of LTA_4_H, are expected to provide potent anti-inflammatory and anti-cancer agents. The aim of the current study is to obtain potential LTA_4_H inhibitors using computer-aided drug design. A hybrid 3D structure-based pharmacophore model was generated based on the crystal structure of LTA_4_H in complex with bestatin. The generated pharmacophore was used in a virtual screen of the Maybridge database. The retrieved hits were extensively filtered, then docked into the active site of the enzyme. Finally, they were consensually scored to yield five hits as potential LTA_4_H inhibitors. Consequently, the selected hits were purchased and their biological activity assessed in vitro against the epoxide hydrolase activity of LTA_4_H. The results were very promising, with the most active compound showing 73.6% inhibition of the basal epoxide hydrolase activity of LTA_4_H. The results from this exploratory study provide valuable information for the design and development of more potent and selective inhibitors.

## 1. Introduction

Leukotriene B4 (LTB4) is a potent chemoattractant and activator of inflammatory cells including neutrophils, eosinophils, macrophages, mast cells, and T cells [1,2,3,4,5,6]. LTB4 plays significant pathological roles in several inflammatory diseases such as inflammatory bowel disease [7,8], rheumatoid arthritis [9,10] and asthma [11,12,13]. Moreover, several studies showed that LTB4 is implicated in cancer development and progression. Elevated levels of LTB4 have been detected in various types of human cancer, where it acts as a key mediator that stimulates cancer cell proliferation [14,15,16]. The biosynthesis of LTB4 is regulated by the action of leukotriene A4 hydrolase (LTA_4_H) in the 5-lipooxygenase (5-LO) pathway of arachidonic acid metabolism [17,18]. LTA_4_H is highly expressed in certain types of human cancers such as lung cancer, thyroid cancer, and skin cancer [19,20,21,22,23,24]. Therefore, inhibition of LTA_4_H/LTB4 pathway should serve as a therapeutic approach in pathological processes, such as inflammation and cancer.

**Figure 1:**
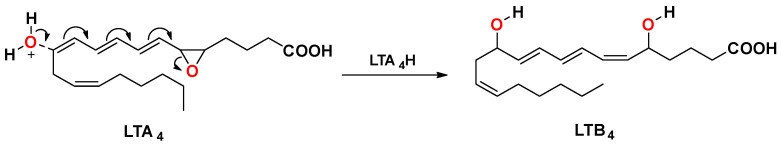
The final rate-limiting step in the biosynthesis of leukotriene B4.

LTA_4_H is a monomeric, cytosolic, zinc metalloenzyme found in an array of human tissues. It is a bifunctional enzyme possessing both epoxide hydrolase and aminopeptidase activities. As an epoxide hydrolase, the enzyme catalyzes the hydrolysis of the unstable epoxide LTA4 into the diol LTB4, which is the final and rate-limiting step in the biosynthetic production of LTB4 (Figure 1) [25,26].

**Figure 2:**
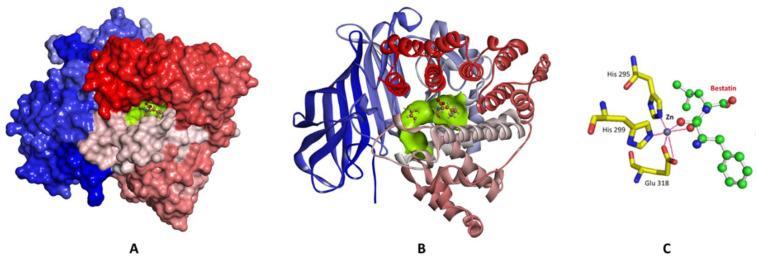
The crystal structure of LTA4H-Bestatin complex (PDB code 1HS6). (**A**) Surface representation of the enzyme with the active site colored green, and the complexed bestatin in balls and sticks. (**B**) Cartoon representation of the enzyme colored N-to-C terminal, blue-to-red respectively. The active site is shown as a green surface, and bestatin in balls and sticks. (**C**) A close-up view of the active site showing the amino acid residues (yellow carbon skeleton) involved in coordinating the Zn ion (gray sphere) and bestatin (green carbon skeleton); coordination interactions with Zn are shown as red lines.

Structurally, LTA_4_H is a protein folded in three domains, *N*-terminal, catalytic, and *C*-terminal domains, packed closely to each other creating a deep cleft [27]. The catalytic domain consists of two lobes; one is mainly α-helical and the other is mixed α/β. A zinc binding site is located between the two lobes where zinc is coordinated to His 295, His 299, and Glu 318. The high-resolution crystal structure of LTA_4_H in complex with the competitive inhibitor bestatin (PDB code 1HS6, resolution 1.95 Å) reveals that the zinc metal is also coordinated with the carbonyl and hydroxyl oxygens of bestatin (Figure 2). In addition to the zinc binding site, LTA_4_H contains a substrate binding pocket which is a hydrophobic cavity ~ 6–7 Å wide and stretches 15 Å deep into the protein. It is a bent and narrow pocket made of hydrophobic amino acids (Figure 2). When bestatin binds to the enzyme, the hydrophobic cavity is occupied by the substrate phenyl ring. Moreover, the substrate binding pocket contains a carboxylate recognition site consisting of Arg563 and Lys565 in which the positive charges in these amino acids make electrostatic interactions with the negative charge of the carboxylate group of bestatin. Understanding the important binding interactions between the enzyme and the substrate should provide valuable information to direct the design of potent therapeutic agents targeting LTA_4_H.

In past decades there have been extensive efforts to identify and design potent LTA_4_H inhibitors using different drug discovery approaches. Several studies describing the discovery and development of LTA_4_H inhibitors have appeared in the literature [20,28,29,30,31,32,33,34,35]. Despite considerable progress, only a few LTA_4_H inhibitors have reached Phase II clinical trials, with only one drug reaching the market, ubenimex^®^ (bestatin) [31,32]. Therefore, there is a growing need for the identification of potent drugs that can inhibit LTA_4_H selectively. In the present study, our efforts focus on the identification of novel competitive inhibitors targeting LTA_4_H using computer-aided drug design. To this end, due to the pronounced effects of bestatin as an anti-cancer drug [22,23] and the availability of the LTA_4_H-bestatin complex crystal structure, we used bestatin binding interactions with LTA_4_H, inferred from the crystal structure, to search for LTA_4_H inhibitors with novel chemotypes as potential anti-cancer agents. The approach used in this study searched for compounds that include zinc chelating moieties which are expected to selectively bind to the zinc atom in the active site of the enzyme.

## 2. Results and Discussion

### 2.1. Structure-Based Pharmacophore Generation

**Figure 3:**
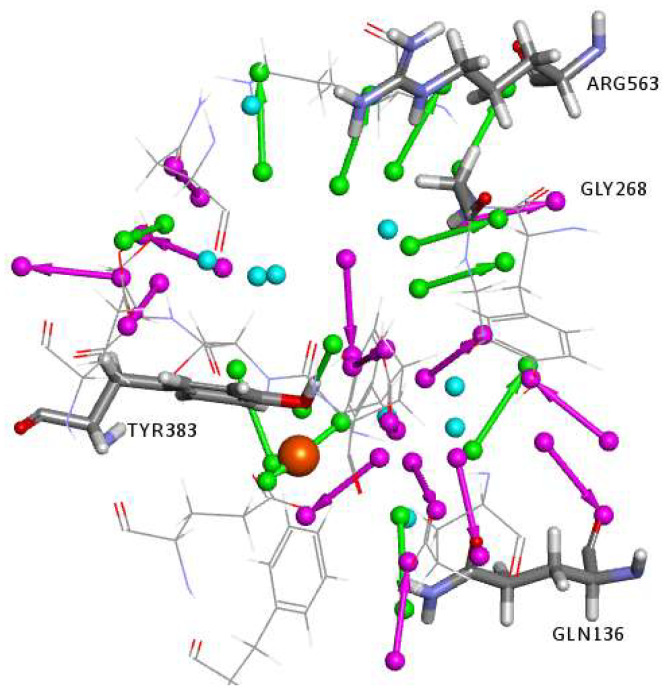
Clustered and edited features obtained from the interaction generation protocol. Location spheres were hidden for clarity. HBD are in magenta, HBA are in green, and the HY are in cyan. The zinc atom is shown as an orange sphere.

Interaction Generation Protocol: The active site in LTA_4_H (PDB code 1HS6) was used to generate a 3D structure-based pharmacophore model to be employed in the virtual screening of small molecules databases, as described in the methods section. The defined binding site was analyzed by applying the Interaction Generation protocol, which uses the Ludi algorithm to generate an interaction map of the binding site. A set of 3D pharmacophoric queries was then derived from the interaction map and were then clustered and edited (Figure 3). This protocol treats the Zn atom as hydrogen bond donors (HBD), therefore the identified hydrogen bond acceptors (HBA) features complementing the HBD feature of the Zn atom were manually replaced by a Zn-binding feature (as will be discussed later).

**Figure 4:**
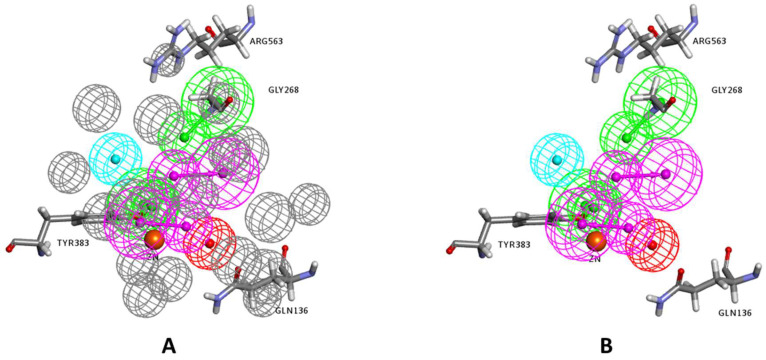
(**A**) The selected pharmacophore model using the Receptor-Ligand Pharmacophore Generation protocol. (**B**) The exclusion volumes are removed for clarity. The feature types are: HBD (magenta), HBA (green), HY (cyan), and PI (red). The zinc atom is shown as an orange sphere.

The Receptor-Ligand Pharmacophore Generation protocol: This protocol was used to generate a pharmacophore based on the LTA_4_H-bestatincomplex (PDB code 1HS6). A set of pharmacophoric features were identified that correspond to the receptor-ligand interactions. The generated pharmacophore models were enumerated and ranked. In this study, 10 pharmacophore models were generated (Table 1). The ranking process is based on measures of selectivity predicted from a Genetic Function Approximation (GFA) model [36]. In this study, model **1** was selected to build the final pharmacophore. Model **1** showed the highest selectivity score and consisted of six features: 2HBA, 2HBD, a HY feature and a positive ionizable (PI) feature (Figure 4).

Generation of the final pharmacophore: Before proceeding in the construction of the pharmacophore, and in order to identify residues within the binding site that may play an important role in ligand binding, different LTA_4_H inhibitor complexes available in the protein data bank were investigated. Eleven crystal structures of LTA_4_H-inhibitor complexes were examined, namely 2R59, 1HS6, 2VJ8, 3B7R, 4DPR, 3U9W, 3FH5, 3FH7, 3FH8, 3FHE, and 3FTS. This investigation revealed that some protein residues within the binding site are important for effective binding since they are involved in intermolecular interactions with the complexed inhibitors in many of the crystal structures. The amino acid residues that were found to frequently interact with complexed inhibitors were Tyr267, Tyr378 (forming π-π hydrophobic interactions in **8** and **6** complexes, respectively), Tyr383, Gln136, Gly268, Gly269 and Arg563 (forming hydrogen bonding and electrostatic interactions in at least four complexes). Therefore, pharmacophoric features mapping these residues, especially the ones forming hydrogen bonding or electrostatic interactions, were given priority to be included in the construction of the final pharmacophore provided they were mapped in any of the two applied structure-based pharmacophore generation approaches mentioned above.

The final pharmacophore was generated as a hybrid of the above two pharmacophores as follows: all identical features in the two pharmacophores were selected, namely: HBA mapping the amide nitrogen of Gly268; and HBA mapping the phenolic OH moiety of Tyr383. In addition, HBD mapping the carbonyl oxygen of the amide side chain of Gln136; and HBA mapping the guanidine moiety of Arg563 obtained from the Interaction Generation protocol were included. Finally, a Zn-binding feature (ZBF) that replaced the HBA mapping the Zn ion in the interaction generation protocol was included. This feature was placed at the tail of the HBA vector pointing to the Zn^2+^ ion (2 Å from the Zn atom) as detailed in previous studies [37,38]. The distance between the Zn ion in a metalloprotein and the chelating heteroatom are important for effective chelation. The median distances between Zn and the chelating atoms O, N, and S were found to be 1.99, 2.05, and 2.28 Å respectively. Ideally, the distance between Zn and the heteroatom (Zn-X) should be within its corresponding median ± 0.1 Å [39].

**Figure 5:**
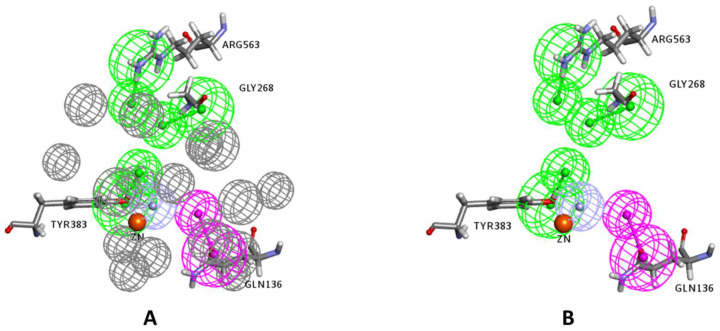
The final generated structure-based pharmacophore model with (**A**) and without (**B**) exclusion constraints (excluded volumes). The feature types are: HBD (magenta), HBA (green), ZBF (light blue), and excluded volumes (gray). The zinc atom is shown as an orange sphere, and key interacting amino acids are labeled and shown in sticks.

The final 3D pharmacophore consists of five features: 1 HBD, 3 HBA, and a Zn-binding feature. Moreover, in order to minimize the number of false positive hits in virtual screening, which are ligands that map the pharmacophore but do not show good docking scores because of steric clashes with protein surface, excluded volumes were added to the final 3D pharmacophore. The excluded volumes define inaccessible regions within the binding site that a ligand may not overlap. The ones generated from the Receptor-Ligand Pharmacophore Generation protocol, which were based on the coordinates of sidechain atoms in vicinity of the query (pharmacophoric) features, were incorporated into the final pharmacophore (Figure 5). For a ligand to be identified as a hit, it should map the query features without bumping the excluded volumes.

### 2.2. Virtual Screening of Commercial Databases

The Maybridge 2017 database, which contains more than 54,000 compounds, was screened for hits that fit the generated pharmacophore. Virtual screening resulted in 674 ligands. Retrieved hits were filtered based on Lipinski’s rule of five and Veber’s rule for drug-like properties. Hits that passed the filtration process were 526. Further filtration based on consideration of the fit values, with a threshold cut-off set to be equal to or greater than 2.0, led to identification of 35 hits that were subjected to molecular docking. The fit value of a retrieved hit is a measure of how well the hits map the pharmacophoric features and whether they deviate from the center of the feature or not; the better the fit, the higher the fit value score. In this study, the fit values of retrieved hits were ranging from ~0 to 4.1. Since we were using a pharmacophore comprised of five features, a perfect hit will get as score of 5. Therefore, we set a cut-off value of greater than or equal to 2 (which is an arbitrary value) to keep hits that are reasonably mapping the pharmacophore and are likely to fit and bind the binding site of the target protein upon docking studies, thereby, enhancing the chances of identifying promising hits.

### 2.3. Molecular Docking and Consensus Scoring

**Figure 6:**
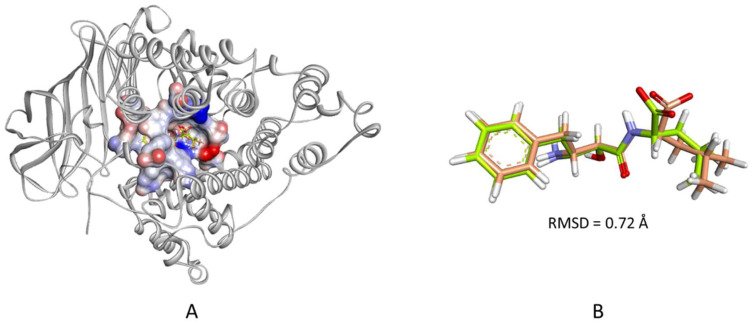
(**A**) The cocrystallized and the redocked bestatin in the LTA_4_H binding site. The protein is shown as white colored cartoon and the biding site as an interpolated charge surface. (**B**) A close-up view of the two superimposed poses. The carbon atoms of the redocked bestatin are colored wheat and those of the cocrystallized pose are colored limon.

Molecular docking of the filtered hits was performed using CDOCKER, and the LTA_4_H-bestatin complex was used to define the binding site as described in the methods section. In docking studies, it is recommended to evaluate the accuracy of the docking algorithm in pose prediction by redocking the cocrystallized ligand to the binding site of the target protein. Once the pose of the redocked ligand agrees with that of the cocrystallized pose, then, it can be taken forward in docking large set of compounds. Therefore, the co-crystallized ligand (bestatin) was extracted from the complex and redocked into the defined binding site. The calculated heavy-atom RMSD between the top ranked redocked pose and that of the crystallized pose was 0.72 Å (Figure 6). Afterwards, the 35 filtered hits were docked into the defined binding site of the enzyme and were found to have-CDOCKER interaction energy scores ranging from 26.89 to 61.81 kcal/mol.

Although docking is an integral part of structure-based drug design, the accuracy of currently available scoring functions remains a major challenge [40]. Nonetheless, a commonly used approach to enhance the accuracy of docking scores is consensus scoring. With consensus scoring, one can identify ligands that score high in more than one scoring function, thereby, leading to an enhancement in hit-rates by balancing errors and deficiencies in individual single scores, thereby, reducing the number of false positives that would be identified using individual scoring functions [41,42,43]. Currently available scoring functions are classified into three groups, force field scoring functions, knowledge-based scoring functions, and empirical scoring functions [44]. In consensus scoring, it is recommended to use 3–4 different scoring functions. The consensus score for a docked ligand is an integer that is equal to its frequency in the top rank percentile (defined by user) for each scoring function. In this study, the docked ligands were rescored using two additional scoring functions, the knowledge-based PMF04 and the empirical PLP2, then, they were consensually scored. The -PMF04 scores ranged from 61.95 to 158.19 kcal/mol, and the -PLP2 scores ranged from 39.34 to 130.88 Kcal/mol. Of the 35 docked hits, only five had a consensus score of three (among the top ranked 30%) in CDOCKER interaction energy, the PMF04, and the PLP2 scoring functions (Table 2). Those five hits were selected as potential inhibitors of the LTA_4_H enzyme and their inhibitory activities experimentally evaluated.

### 2.4. Inhibition Assay of Hydrolase Activity of LTA_4_H

The five compounds listed in Table 2 were purchased and their biological activities assessed in vitro against the epoxide hydrolase activity of human LTA_4_H at a concentration of 25 µMand their percent LTA_4_H inhibition measured relative to the uninhibited enzyme. As shown in Table 3, the inhibitory activities of the tested compounds were quite promising, with the % inhibition of LTA_4_H ranging from 8 to 73.6%.

The above results reveal weak to very good inhibitory activity of the identified compounds with the most active compound, RH00633, showing 73.6% inhibition of enzyme activity. Based on these promising results, further studies will to be done to expand the search for other potential inhibitors with different chemotypes that would facilitate the optimization process toward designing a drug-like compound with better selectivity and potency.

**Figure 7:**
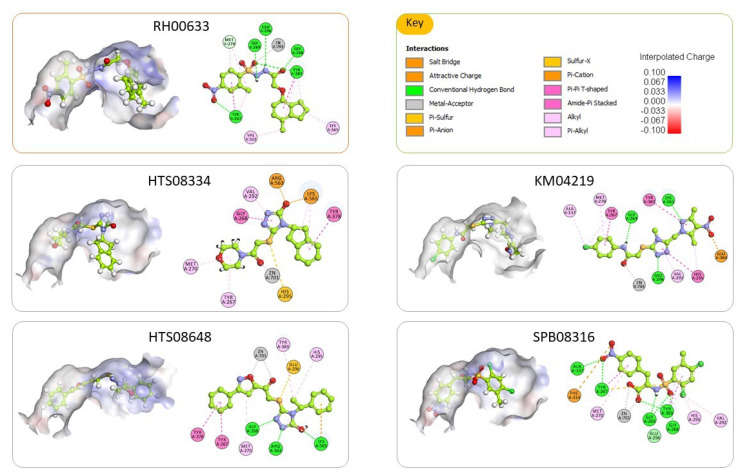
The top ranked docked pose of the five tested compounds. For each compound, there is a 3D depiction of the docked pose showing its orientation within the binding site and a 2D interaction map with the enzyme. The binding site is shown as an interpolated charge surface, and the docked compounds are shown as balls and sticks with carbons colored limon. Residues in the 2D interaction map are shown as disks and colored according to the type of interactions with the ligand.

The top ranked docked pose of the five tested compounds and their 2D interaction maps are shown in Figure 7. For the most active compound (RH00633), the docked pose reveals that it occupies the binding pocket and establishes numerous interactions with surrounding amino acid residues. The major interactions are through coordination with the Zn^2+^ ion; hydrogen bonding with Tyr 267, Gly 268, Gly 269, Glu 296, and Tyr 383; and many hydrophobic interactions, mainly pi-pi stacking with Tyr 267 and Tyr 383. Those numerous and effective interactions explain the shown inhibitory effect of this compound.

Similarly, the other compounds are well-fitting the binding site and are forming numerous interactions with the surrounding amino acid residues. In all cases, they were forming metal acceptor interaction with Zn^2+^ ion. Besides, they were forming many hydrogen bonds and hydrophobic interactions with the previously identified amino acid residues that are deemed vital for effective ligand binding such as Gln136, Tyr 267, Gly 268, Gly 269, Tyr378, Tyr 383 and Arg563.

## 3. Materials and Methods

### 3.1. Materials

Preparation of the starting leukotriene A4 hydrolase structure was performed using Discovery Studio (DS) 2017 from Biovia^®^ (formerlyAccelrys^®^) Software Inc. (San Diego, CA, USA) [45]. Pharmacophore modeling and virtual screening were performed using DS. Docking of proposed inhibitors was performed using CDOCKER within DS [46]. Presentation quality images was generated using DS. Leukotriene A4 Hydrolase (human recombinant), LTA4 methyl ester, LTB4 ELISA kit and Ultra-Pure water were purchased from Cayman Chemical (Ann Arbor, MI, USA). The selected compounds were purchased from Maybridge Chemical Holdings Ltd., UK (which is a brand of Thermo Fisher Scientific, Waltham, MA, USA) via local vendors.

### 3.2. Methods

#### 3.2.1. Preparation of the LTA_4_H Enzyme

The structural model of the leukotriene A4 hydrolase was prepared using DS, where the initial coordinates for the enzyme were retrieved from the Protein Data Bank (entry code 1HS6, resolution of 1.95Å) which corresponds to leukotriene A4 hydrolase in complex with bestatin [27]. The PDB file was checked for missing loops, alternate conformations and incomplete residues using *Protein Report*. The *Prepare Protein Tool* summarizes key information about the protein structure including: comparison of the actual sequence with the PDB SEQRES records; residues with alternate conformations; a list of incomplete or invalid residues; active site definitions; and an annotation of any gaps in the structure. Then, the structure was cleaned and prepared using the *Prepare Protein* protocol which prepares proteins for input into other protocols by performing tasks such as inserting missing atoms in incomplete residues, modeling missing loop regions, deleting alternate conformations (disorder), standardizing atom names, and protonating titratable residues using predicted pKs. Finally, it was typed using the *simulation tools* by applying the CHARMm force field.

#### 3.2.2. Structure-Based Pharmacophore Generation

The active site of the enzyme was used to generate a 3D structure-based pharmacophore (SBP) model to be used in virtual screening of small molecules databases. Two approaches were used to generate this pharmacophore, namely; the Interaction Generation and Receptor-Ligand Pharmacophore Generation protocols.

The Interaction Generation Protocol: This protocol applies the Ludi algorithm which generats an interaction map by enumerating interaction points (sites) within a defined protein binding site that are important for ligand binding. For each atom or functional group of the protein that is capable of participating in a nonbonded contact, a set of interaction points is generated which encompasses the range of suitable positions for a ligand atom or functional group involved in the putative interaction. The generated interaction map consists of hydrogen bond acceptor, hydrogen bond donor, and hydrophobes, which are then converted to pharmacophoric features [47,48]. To run the protocol, the binding site was defined with a sphere that covered all important amino acid residues. The sphere was created around the cavity that hosts the bound ligand using the Define and Edit Binding Site tool. The sphere was expanded from 7.61 to 9 Å in order to encompass all residues in the binding site that maybe of relevance to ligand binding. Then, the protocol was employed, using default parameters. The identified hydrogen bond acceptors (HBA), hydrogen bond donors (HBD), and hydrophobic (HY) features were then averaged and edited using the Edit and Cluster Pharmacophore Features tool.

The Receptor-Ligand Pharmacophore Generation protocol: In this protocol, the prepared LTA_4_H-bestatin complex was used to generate a set of selective pharmacophore models. The protocol was applied using default parameters.

The final pharmacophore was generated as a hybrid of pharmacophores generated using the above two approaches. To account for steric interactions with the protein, excluded volumes were added to the generated pharmacophore. All exclusion volumes generated from the Receptor-Ligand Pharmacophore Generation protocol were incorporated in the final pharmacophore and those that were overlapping with the tolerance spheres of the pharmacophoric features were removed.

#### 3.2.3. Virtual Screening of Commercial Databases

The generated pharmacophore was used in virtual screening of the Maybridge database using the Best Flexible Search method in the Search 3D Database protocol. Retained hits were then filtered based on Lipinski’s rule of five and Veber’s rule of drug-like properties and consideration of fit values. Hits that passed all filtration criteria were selected for molecular docking.

#### 3.2.4. Molecular Docking

Molecular docking of the filtered hits was performed using CDOCKER (CHARMm-based DOCKER) within DS, which is a grid-based molecular dynamic docking algorithm. This algorithm is a rigid-flexible type docking algorithm, where it treats the protein as a rigid molecule but accounts for full ligand flexibility via high temperature molecular dynamics followed by random rotations; and to refine the docked poses it performs a final minimization or simulated annealing step. The generated poses are then scored based on CHARMm energy (interaction energy plus ligand strain) and the interaction energy alone. The top ranked poses based on interaction energy (the most negative, favorable interaction) are retained [46]. The same sphere-defined binding site used for *Interaction Generation Protocol* was used for docking purposes. Then CDOCKER protocol was employed using default parameters.

To consensually score the docked ligands, they were rescored using different scoring functions available in DS; namely PMF04 (a knowledge-based scoring function), and PLP2 (an empirical scoring function). This was carried out using the Score Ligand Poses protocol. Then, a consensus score based on a consensus percentage of 30 was calculated using the Consensus Score protocol.

#### 3.2.5. In Vitro Enzyme Inhibition Assay

Preparation of Substrate: LTA4 was prepared through the hydrolysis of LTA4 methyl ester (Cayman Chemical) in a degassed solution of 50 mM NaOH (20%, *v*/*v*) in cold acetone under an inert atmosphere of nitrogen at 25 °C for 60 min. The resulting LTA4 solution was directly diluted using a freshly prepared buffer (10 mM sodium phosphate, pH 7.4, 4 mg/mL BSA, 2.5% *v*/*v* DMSO). The LTA4 solution was freshly prepared prior to use.

Epoxide Hydrolase Assay: To determine the effect of the selected compounds on the epoxide hydrolase activity of LTA_4_H, 300 ng of enzyme was incubated with test compounds (final concentration of 25 µM) in 180 µL of reaction buffer (10 mM sodium phosphate, pH 7.4, 4 mg/mL BSA, 2.5% *v*/*v* DMSO) for 15 min at 37 °C. Then 20 µL of the LTA4 was added (final concentration of 150 nM, final volume of 200 µL) and incubated for another 10 min at 37 °C. Then the assay was terminated by diluting 20-fold in assay buffer. The amount of LTB4 produced was quantified in the diluted samples by a commercially available LTB4 ELISA kit (Cayman Chemical) [49].

## 4. Conclusions

In this study, a hybrid 3D structure-based pharmacophore model was generated based on the crystal structure of LTA_4_H-bestatincomplex. More than 54,000 compounds in the Maybridge database were virtually screened using the generated pharmacophore to identify potential inhibitors of the target enzyme. The retained hits were extensively filtered and promising compounds docked into the binding site of the enzyme in order to have an estimation of their binding affinity to aid in the selection of potential inhibitors.

To further refine the number of retained hits, prioritize them, and to account for the shortcomings of individual scoring function, the docked hits were rescored using different scoring functions followed by consensus scoring. Based on consensus scoring five hits were selected as potential LTA_4_H inhibitors. The five selected hits were purchased and their biological activity assessed in vitro against the epoxide hydrolase activity of LTA_4_H, which showed very good inhibitory activity. The most active compound will be used as a lead compound for further optimization. The LTA_4_H inhibitory ability of the lead compound and its analogs will be assessed in cell-free assays and against different cell lines.

## Figures and Tables

**Table 1:** The ten pharmacophores generated using the Receptor-Ligand Pharmacophore Generation protocol.

Pharmacophore Summary			
Pharmacophore	Number of Features	Feature Set	Selectivity Score
Pharmacophore_01	6	AADDHP	12.549
Pharmacophore_02	5	AADDP	11.034
Pharmacophore_03	5	ADDHP	11.034
Pharmacophore_04	5	ADDHP	11.034
Pharmacophore_05	5	AADHP	10.120
Pharmacophore_06	5	AADHP	10.120
Pharmacophore_07	5	AADDH	9.640
Pharmacophore_08	4	ADDP	9.519
Pharmacophore_09	4	ADDP	9.519
Pharmacophore_10	4	DDHP	9.519

**Table 2:** Selected hits that are potential inhibitors of the LTA_4_H enzyme.

Code	Structure	Fit Value *	Consensus Score
KM04219	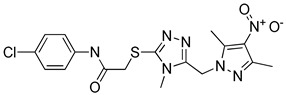	3.542	3
SPB08316	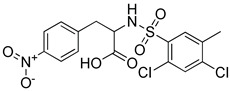	3.084	3
RH00633	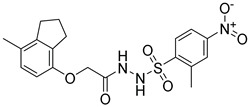	2.432	3
HTS08334	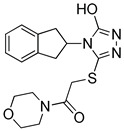	2.144	3
HTS08648	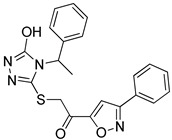	2.052	3

*A measure of how well retrieved hits map the pharmacophoric features.

**Table 3:** Inhibitory activity of the selected hits.

Compound Code	% of LTA_4_H Inhibition (at 25 μM) ^a^
KM04219	44.5
SPB08316	51.9 ± 0.84
RH00633	73.6 ± 0.25
HTS08334	45 ± 17.1
HTS08648	8 ± 14.6

^a^ Compounds were tested in duplicate and are presented as mean ± standard error of the mean.
